# The role of psychosis and clozapine load in excessive checking in treatment-resistant schizophrenia: longitudinal observational study

**DOI:** 10.1192/bjp.2024.30

**Published:** 2024-05

**Authors:** Emilio Fernandez-Egea, Shanquan Chen, Estela Sangüesa, Patricia Gassó, Marjan Biria, James Plaistow, Isaac Jarratt-Barnham, Nuria Segarra, Sergi Mas, Maria-Pilar Ribate, Cristina B. García, Naomi A. Fineberg, Yulia Worbe, Rudolf N. Cardinal, Trevor W. Robbins

**Affiliations:** Cambridgeshire and Peterborough NHS Foundation Trust, Cambridge, UK; Department of Psychiatry, University of Cambridge, UK; and Behavioural and Clinical Neuroscience Institute, University of Cambridge, UK; Department of Psychiatry and Division of Psychology and Language Sciences, University College London, UK; Faculty of Health Sciences, Universidad San Jorge, Spain; Department of Basic Clinical Practice, University of Barcelona, Spain; Institut d'Investigacions Biomèdiques August Pi i Sunyer (IDIBAPS), Barcelona, Spain; and Centro de Investigación Biomédica en Red de Salud Mental (CIBERSAM), Madrid, Spain; Behavioural and Clinical Neuroscience Institute, University of Cambridge, UK; and Department of Psychology, University of Cambridge, UK; Cambridgeshire and Peterborough NHS Foundation Trust, Cambridge, UK; Cambridgeshire and Peterborough NHS Foundation Trust, Cambridge, UK; Medical Sciences Division, University of Oxford, UK; Hertfordshire Partnership University NHS Foundation Trust, Welwyn Garden City, UK; and School of Life and Medical Sciences, University of Hertfordshire, UK; Department of Neurophysiology, Sorbonne Université, France; Department of Neurophysiology, Saint-Antoine Hospital, Paris, France; and Institute du Cerveau et de la Moelle Epinière, Paris, France; Cambridgeshire and Peterborough NHS Foundation Trust, Cambridge, UK; and Department of Psychiatry, University of Cambridge, UK

**Keywords:** Habit formation, clozapine, treatment-resistant schizophrenia, serotonin, compulsion

## Abstract

**Background:**

A significant proportion of people with clozapine-treated schizophrenia develop ‘checking’ compulsions, a phenomenon yet to be understood.

**Aims:**

To use habit formation models developed in cognitive neuroscience to investigate the dynamic interplay between psychosis, clozapine dose and obsessive–compulsive symptoms (OCS).

**Method:**

Using the anonymised electronic records of a cohort of clozapine-treated patients, including longitudinal assessments of OCS and psychosis, we performed longitudinal multi-level mediation and multi-level moderation analyses to explore associations of psychosis with obsessiveness and excessive checking. Classic bivariate correlation tests were used to assess clozapine load and checking compulsions. The influence of specific genetic variants was tested in a subsample.

**Results:**

A total of 196 clozapine-treated individuals and 459 face-to-face assessments were included. We found significant OCS to be common (37.9%), with checking being the most prevalent symptom. In mediation models, psychosis severity mediated checking behaviour indirectly by inducing obsessions (*r* = 0.07, 95% CI 0.04–0.09; *P* < 0.001). No direct effect of psychosis on checking was identified (*r* = −0.28, 95% CI −0.09 to 0.03; *P* = 0.340). After psychosis remission (*n* = 65), checking compulsions correlated with both clozapine plasma levels (*r* = 0.35; *P* = 0.004) and dose (*r* = 0.38; *P* = 0.002). None of the glutamatergic and serotonergic genetic variants were found to moderate the effect of psychosis on obsession and compulsion (SLC6A4, SLC1A1 and HTR2C) survived the multiple comparisons correction.

**Conclusions:**

We elucidated different phases of the complex interplay of psychosis and compulsions, which may inform clinicians’ therapeutic decisions.

A significant proportion of people with schizophrenia develop obsessive–compulsive symptoms (OCS). Some experience an obsessive–compulsive disorder (OCD) after remission of psychosis, whereas others enter an intermediate state combining psychosis and OCS.^1^ However, there is no clear understanding of this phenomenon.

## Schizophrenia and OCS/OCD

OCS and OCD are common among those with schizophrenia, with prevalence increasing from 12.5% in individuals in an at-risk mental state for psychosis to 25% in early schizophrenia and up to 47% in clozapine-treated patients.^2^ There is a plausible biological overlap between schizophrenia with OCS and classic OCD. Both feature over-activation of the orbitofrontal cortex^3^ and exhibit similar traits of cognitive inflexibility, reduced processing speed and memory deficits.^4^ Further, individuals with schizophrenia with OCS share a genetic background with OCD. Specifically, single nucleotide polymorphisms (SNPs) and other genetic variants in the glutamate pathway, such as SLC1A1 (glutamate transporter) and GRIN2B (glutamate receptor), are associated with OCS in people with clozapine-treated schizophrenia.^5^ However, associations with serotonergic (5-HT) pathways have not been explored, despite the role of SLC6A4 (a serotonin transporter), HTR2A and HTR2C (serotonin receptors) in OCD.^6^ Some authors advocate for including schizo-obsessive disorder as a schizophrenia subtype in which pre-existing OCS or OCD are unmasked after psychosis remission.^4^ However, this hypothesis is undermined by work showing a correlation between OCS and psychosis severity.^5^ Others argue that *de novo* OCS in schizophrenia are an antipsychotic-related event.^1^ Epidemiologically, *de novo* OCS are over-represented in patients treated with clozapine, olanzapine or risperidone when compared with patients prescribed aripiprazole, amisulpride or haloperidol.^6^ This likely reflects these drugs’ different 5-HT_2A/2C_ receptor affinity. OCS are also associated with higher clozapine dose and length of treatment and clozapine plasma levels,^7^ suggesting a dose-dependent relationship. This is challenged, however, in the literature, with some suggesting that these findings are confounded by the higher antipsychotic doses typically required by those with psychosis of greater severity.^8^

## Using the habit model to understand the interplay between psychosis, OCS and clozapine

We have previously identified that, in clozapine-treated patients, psychosis severity and length of treatment are distinct risk factors for obsessions and checking compulsions respectively.^9^ Most studies show a predominance of compulsions over obsessions in this group, with excessive checking being the most frequently reported repetitive behaviour.^10^ Informed by models of habit formation developed in cognitive neuroscience, we conceptualised these checking compulsions as arising as the by-product of psychosis. Specifically, repetition in the context of a diminished ability to consider an action's outcome may lead to the automatisation of behaviour (‘habit formation’). Further, decreased 5-HT activity may enhance habit development.^11^ We have applied this framework to hypothesise a two-phase model of OCS and OCD development. First, an initial phase of checking as a goal-directed behaviour may occur due to psychotic hypervigilance. After remission of psychosis, achieved with antipsychotic treatment, a second ‘habit’ phase may occur in which clozapine ameliorates psychosis but promotes checking compulsions via serotonin antagonism in those vulnerable.

In the present study, we investigated the dynamic interplay between psychosis, clozapine dose and OCS. We aimed to test this two-phase hypothesis using a large cohort of clozapine-treated patients assessed longitudinally. Specifically, we hypothesised that:

- checking compulsion is related to psychosis severity
- checking severity correlates with clozapine plasma levels in those in remission from psychosis.

We also explored the moderating effects of specific genetic variants on the development of psychosis-related obsessions and compulsions.

## Method

### Study design and setting

This naturalistic longitudinal observational study used anonymised electronic records gathered by Cambridgeshire and Peterborough NHS Foundation Trust (CPFT). CPFT is the primary public mental healthcare provider for approximately 890 000 people in a mixed urban/rural area in the East of England, UK.

### Ethics, participants and electronic records

The authors assert that all procedures contributing to this work comply with the ethical standards of the relevant national and institutional committees on human experimentation and with the Helsinki Declaration of 1975, as revised in 2008.

We used the Clinical and Research Database (CRD) for Persistent Schizophrenia under NHS Research Ethics Committee (REC) approvals (ref. 13/EE/0121; 18/EE/0239). This database contains anonymised routine clinical data from the CPFT Clozapine Clinic.

Extracted data maintained patient anonymity by removing all identifiable data. All clinical assessments in the CRD were performed by an experienced psychiatrist (E.F.E.) and the database also contained self-rated measures that the patients completed during routine clinical appointments. This study covers information from 24 August 2012 to 31 December 2022. The CRD includes 3126 face-to-face assessments of 254 patients taking clozapine. For this study, we included only assessments that had a standardised evaluation of OCS and scores on the positive subscale of the Positive and Negative Syndrome Scale (PANSS-positive) (see below).

### Routine clinical assessments

All annual Care Programme Approach (CPA) assessments in the electronic record include sociodemographic data (age, gender, age at illness onset, clozapine start date), a review and confirmation of prescribed medication (with dose), current smoking habit (average number of cigarettes per day), alcohol use (average number of alcohol units per week) and latest clozapine and norclozapine plasma levels results (including date of test). All patients are evaluated on all the scales.

For this study, relevant psychopathology scales included in the CRD were the Obsessive–Compulsive Inventory – Revised (OCI-R), self-reported annually from 2016 (and completed independently of other scales), and the PANSS-positive, rated every 2 years since 2017.

### Psychopathology scales

Severity of OCS was measured using the OCI-R,^12^ an 18-item self-reported measure featuring six subscales: washing, checking, ordering, obsessing (e.g. having obsessional thoughts), hoarding and mental neutralising. The impact of each item on a respondent's function is reported via a 5-point scale, ranging from ‘not at all’ (0) to ‘extremely’ (4). The total score, therefore, ranges from 0 to 72, with higher scores indicating greater OCS severity. As in previous work,^9^ we considered a total score ≥21, or ≥5 on any subscale, as clinically significant.

Psychosis severity was measured using the positive subscale (items P1–P7) of the PANSS^13^. The PANSS is a clinician-rated scale that includes 7 items referring to psychotic (‘positive’) symptoms rated on a 7-point scale (1 absent, 7 extreme). Patients were considered in remission from psychosis when none of these items scored ≥3, as the main goal of the study was to explore whether active symptoms (PANSS score of ≥3) were associated with OCS/OCD.

### Assessment of clozapine load

We used blood clozapine levels as a measure of clozapine load, rather than clozapine dose, as dose (typically ranging from 75–900 mg/day) is an inaccurate measure of clozapine load.^14^ This is because clozapine metabolism is influenced by various factors, including medication adherence, gender, cytochrome polymorphisms, concurrent medication and smoking habits. Blood levels, therefore, represent a more precise measure.

### Genetics of clozapine-induced OCS

One hundred patients of the final sample of 196 also consented to participate in the ethically approved ‘Genetics of common clozapine-induced side effects’ study (REC reference 18/NW/0581). The study aimed to replicate previously described genetic variants associated with clozapine side-effects, including OCD and metabolic complications.

Specifically, we explored genetic variants in the serotonin pathway, including single nucleotide polymorphisms (SNPs): SLC6A4 (rs4795541, rs25531), HTR2A (rs6313, rs6314) and HTR2C (rs3813929, rs1414334). Variants within the glutamate pathway were also included: SLC1A1 (rs2228622) and GRIN2B (rs890). Samples were collected during routine blood monitoring at the clozapine clinic, using Whatman^®^ FTA^®^ (Flinders Technology Associates) cards by Sigma-Aldrich, allowing storing, transporting, stability and DNA purification from samples at room temperature.

Genotyping was conducted at San Jorge University in Zaragoza and in the Pharmacology Unit of Barcelona University Medical School, both in Spain. Using a paper puncher sterilised with alcohol and flame, 3 mm discs of dried blood were obtained from the card. DNA was extracted following the manufacturer's instructions. The concentration and quality of DNA were measured spectrophotometrically using a NanoDrop™ 2000 (Thermo Fisher Scientific, Surrey, UK). Genetic variants of the SLC6A4 gene, rs4795541 (also known as 5-HTTLPR) and rs25531, were genotyped using an MJ Mini™ thermal cycler (Bio-Rad, Hercules, CA, USA) following polymerase chain reaction (PCR) and restriction fragment length polymorphism (RFLP) conditions described previously.^9^ The CFX Connect Real-Time PCR System (Bio-Rad, Hercules, CA, USA) was used to genotype rs6313 and rs6314 (HTR2A) with predesigned rhAmp™ SNP Genotyping Assays (Integrated DNA Technologies, Coralville, IA, USA). The polymorphisms rs3813929 and rs1414334 (HTR2C), rs2228622 (SLC1A1) and rs890 (GRIN2B) were genotyped using an MJ Mini™ thermal cycler (Bio-Rad, Hercules, CA, USA) following PCR and RFLP conditions previously described.

The Strengthening the Reporting of Observational Studies in Epidemiology (STROBE) guidelines checklist was followed.

### Statistical analysis

For basic description, categorical variables are reported in the format ‘*n* (%)’ and continuous variables in the format ‘mean (s.d.)’.

Pearson correlation coefficients were used to measure the strength and direction of associations between continuous variables. To account for multiple comparisons, we applied a Bonferroni correction.

A multi-level mediation model assessed the longitudinal association between psychosis severity and checking compulsions, directly and indirectly via obsessing. This is the preferred method for exploring longitudinal changes in samples where the time between assessments is not fixed, as in our study. Psychosis severity (measured by the PANSS-positive subscale) was included as both a fixed-effect and a (per-participant) random-effect variable, and the duration of clozapine treatment was controlled for, as in previous similar studies. Further analyses used PANSS-positive items associated with reality distortion (items P1, P3 and P6).

For assessing the effect of clozapine load on psychosis severity, we selected a subgroup of participants to avoid factors confounding previous research. These individuals (a) were on clozapine monotherapy (no other antipsychotic or antidepressant) for more than a year, (b) were in remission from psychosis and (c) had plasma levels taken within 28 days of the OCS evaluation (without intervening medication changes) that were within the upper limit of the therapeutic range (0.6 mg/L or 600 ng/mL).

Multi-level moderation models were also conducted to explore whether specific genetic variants moderated the association between psychosis severity and checking compulsions via obsessions. The models were fitted with psychosis severity and genetic variant as both fixed-effect and (per-participant) random-effect variables. Duration of clozapine use was controlled for. This was performed in a subgroup of patients for whom genetic data were available (*n* = 97, with 235 face-to-face assessments).

All statistical analyses were performed using R (version 3.5.0 for Windows), including the packages lmerTest (version 3.1-2) and Mediation (version 4.5.0).

## Results

### Sample description

The final sample consisted of 196 clozapine-treated patients and 459 OCI-R/PANSS-positive pair assessments, each participant being followed for an average of 2.7 years. Table 1 shows key sociodemographic and clinical variables at baseline: 74 participants (37.9%) had an OCI-R score at or above the OCD cut-off of 21. Obsessing and checking compulsions were the most common OCS. Among those in remission from psychosis (*n* = 60 out of 196), 25 (12.8% of the total) exhibited significant checking behaviours (indicated by a score >4 on the checking subscale).

**Table 1:** Baseline sociodemographic and clinical characteristics of the participants (*n* = 196)

Variable	*n* (%) or mean (s.d.)
Age at baseline, years	47.44 (10.52)
Age at first episode of psychosis , years	22.54 (6.86)
Age at starting clozapine, years	31.41 (8.83)
Gender (male)	155 (79.1%)
Follow-up, years	2.71 (1.89)
Number of face-to-face assessments	
1	47 (24.0%)
2	59 (30.1%)
3	68 (34.7%)
>4	22 (11.2%)
OCI-R total score ≥21	74 (37.9%)
Factor 1: washing ≥5	27 (13.8%)
Factor 2: obsessing ≥5	82 (42.1%)
Factor 3: hoarding ≥5	53 (27.2%)
Factor 4: ordering ≥5	47 (24.1%)
Factor 5: checking ≥5	85 (43.6%)
Factor 6: neutralising ≥5	39 (20.0%)
Duration of clozapine, years	17.51 (8.91)
Clozapine dose, mg/day	335.8 (145.0)
PANSS-positive (psychosis) score	12.59 (4.50)
OCI-R total	18.44 (13.55)
Factor 1: washing	1.73 (2.40)
Factor 2: obsessing	4.22 (3.49)
Factor 3: hoarding	3.06 (2.77)
Factor 4: ordering	2.56 (2.77)
Factor 5: checking	4.49 (3.50)
Factor 6: neutralising	2.37 (3.06)

OCI-R, Obsessive–Compulsive Inventory – Revised. PANSS, Positive and Negative Syndrome Scale.

Thirty-five participants (17.9%) exhibited negligible OCS (total score <5 and checking factor <2) after 5 years or more on clozapine treatment. The Supplementary material available at https://doi.org/10.1192/bjp.2024.30 expands on the details of the prevalence of OCS severity by psychosis remission.

### The role of psychosis and reality distortion in OCS

Psychotic symptoms significantly correlated with overall OCS severity and with the obsessing and checking compulsion subscales of the OCI-R (Table 2). This association was significantly stronger for obsessing (*r* = 0.419) than compulsion (*r* = 0.116). Table 2 also shows the associations with the individual psychotic symptoms.

**Table 2:** Correlation between obsessive–compulsive symptoms (OCS) and psychotic symptoms^a^

	PANSS-positive	Delusions (P1)	Disorganisation (P2)	Hallucinations (P3)	Excitement (P4)	Grandiosity (P5)	Persecution (P6)	Hostility (P7)
OCI-R total score	***r* = 0.289** ***P* < 0.001**	***r* = 0.264** ***P* < 0.001**	*r* = 0.025 *P* = 0.592	***r* = 0.297** ***P* < 0.001**	*r* = 0.027 *P* = 0.569	*r* = 0.009 *P* = 0.852	***r* = 0.333** ***P* < 0.001**	*r* = −0.061 *P* = 0.194
OCI-R Obsessing	***r* = 0.419** ***P* < 0.001**	***r* = 0.393** ***P* < 0.001**	*r* = 0.084 *P* = 0.074	***r* = 0.450** ***P* < 0.001**	*r* = −0.071 *P* = 0.131	*r* = −0.006 *P* = 0.902	***r* = 0.440** ***P* < 0.001**	*r* = −0.038 *P* = 0.419
OCI-R Checking	*r* = 0.116 *P* = 0.014	*r* = 0.113 *P* = 0.016	*r* = −0.056 *P* = 0.240	***r* = 0.165** ***P* < 0.001**	*r* = 0.002 *P* = 0.974	*r* = −0.044 *P* = 0.356	***r* = 0.181** ***P* < 0.001**	*r* = −0.093 *P* = 0.049

PANSS, Positive and Negative Syndrome Scale; OCI-R, Obsessive–Compulsive Inventory – Revised.

a. OCS were measured using the OCI-R total score and OCI-R Obsessing and Checking subscales. Psychosis was measured using the PANSS positive subscale (*n* = 457) and individual PANSS positive symptoms (sum of P1 to P7). Bold indicates significance after correction for multiple comparisons (α = 0.05/24 = 0.002).

**Fig. 1:**
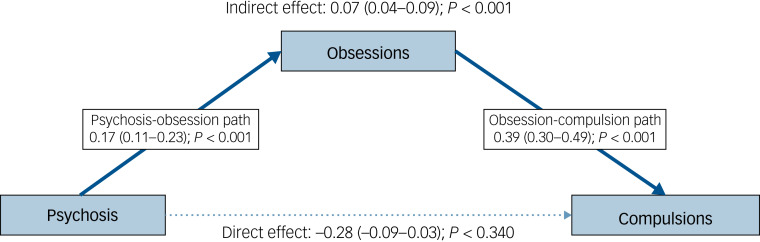
Obsessive–compulsive symptoms (OCS) and psychosis: mediation model exploring causality (*n* = 195, with 459 face-to-face assessments). Psychosis was measured using the positive subscale of the Positive and Negative Syndrome Scale (PANSS). Effects are reported with 95% confidence intervals.

The effect of psychosis on checking behaviour was indirect, mediated by obsessional symptoms. No direct effect of psychosis on checking compulsions was identified (Fig. 1). The length of clozapine treatment was controlled for in this model. In further models (Supplementary Figs 1 and 2), an indirect effect of psychosis on compulsion was found only for participants with active paranoid/psychotic symptoms (*n* = 198: those for whom the sum of scores on PANSS items P1 + P3 + P6 > 4). Similar results were found when additional confounders, including clozapine dose, were included in the models.

### The role of clozapine on the persistence of excessive checking after psychosis remission

**Fig. 2:**
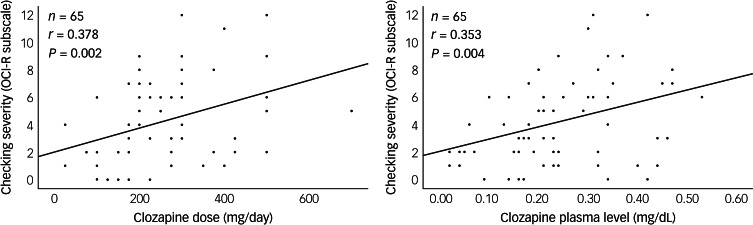
Correlation of checking severity with clozapine dose and plasma levels in the subgroup (*n* = 65) on clozapine monotherapy and in remission from psychosis. Clozapine plasma levels were taken within 28 days of the assessment and with no intervening medication changes. OCI-R, Obsessive–Compulsive Inventory – Revised.

Figure 2 shows the significant correlations between checking compulsions and clozapine dose (*n* = 65; *r* = 0.378, *P* = 0.002), clozapine plasma level (*r* = 0.353; *P* = 0.004) and norclozapine level (*r* = 0.270; *P* = 0.030). There was no correlation between obsessing and either clozapine dose or levels (*P* > 0.21).

Within this group of patients in remission, we compared those with excessive checking (OCI-R checking subscale score >4; *n* = 28) with those without checking (*n* = 37). Those with excessive checking had a higher clozapine dose (*t* = 2.956; *P* = 0.04), higher clozapine levels (*t* = 2.973; *P* = 0.04) and higher norclozapine levels (*t* = 2.363; *P* = 0.021).

Across the whole sample (thus, including those with any severity of psychosis or non-monotherapy; *n* = 313), there were no significant correlations between checking severity and clozapine dose (*r* = 0.071; *P* = 0.213) or plasma level (*r* = 0.021; *P* = 0.709).

### Genes moderating psychosis-to-obsession and obsession-to-compulsion effects

We found the association of psychosis with obsessions to be moderated by serotonergic genes in uncorrected tests, but this did not survive correction for multiple comparisons. In the uncorrected tests, genotype GC for HTR2C (rs1414334) moderated the effect, and genotype GA SLC6A4 (rs25531) and genotype AA for SLC1A1 (rs2228622) moderated the effect from obsession to compulsion, but none of the other SNPs studied did. Supplementary Table 1 details the eight SNPs studied.

## Discussion

We found that OCS are common in clozapine-treated patients and are associated with both psychosis severity and clozapine load, albeit at different phases. Mediation analyses suggested that psychosis severity generates checking behaviour indirectly by inducing obsessions. After remission from psychosis, checking compulsion correlated with clozapine plasma levels. We also found indications that serotonergic and glutamatergic variants might moderate the effect of psychosis on obsessions and compulsions, although our sample was too small for these findings to be considered conclusive.

### Limitations

This study has some limitations. First, its naturalistic design is inferior to an experimental study. Experimental methodologies, however, are not feasible here, as the potentially lethal side-effects of clozapine generally prohibit its chronic administration to healthy volunteers. Long-term longitudinal studies in people with schizophrenia after clozapine initiation could provide a more detailed characterisation, but the latency of the onset of OCS (up to a decade) may render such studies impractical. Given this, we consider this study's large sample and longitudinal follow-up to represent a balance between practical feasibility and rigour. Second, OCS were evaluated using a self-rated scale and further work might benefit from replication using a clinician-rated scale such as the Yale–Brown Obsessive Compulsive Scale (Y-BOCS). Again, however, this may be impractical in a clinical environment. Third, the patient sample included in this study's genetic analyses was small (*n* = 97), and these findings should therefore be taken as exploratory and in need of replication.

### Interpretation of findings and comparison with previous research

Our results broadly align with previous research regarding OCS prevalence (here, 37.9%) and the predominance of checking and obsessing symptoms. Our sample is representative of a typical clozapine-treated cohort, with male predominance (79%), an average prescribed clozapine dose of ~330 mg/day and a typical treatment length (~17 years). Importantly, however, this study's sample size (196 participants), longitudinal nature and volume of standardised OCS and PANSS-positive assessments (457 face-to-face assessments) are more extensive than any previous research.

This work builds on a hypothesis established *a priori* and developed during our previously published cross-sectional publication. We have conceptualised clozapine-associated OCS as a dynamic phenomenon that fluctuates in intensity according to psychosis severity, in line with recent work by Schirmbeck and colleagues,^15^ and clozapine load. We uncoupled OCS into its two main components (obsessions and compulsions) and applied a mediation model that identified an association of psychosis severity (particularly the severity of reality distortion symptoms such as delusions, hallucinations or suspiciousness/persecutory beliefs) with obsessive thoughts. Obsessive thoughts, in turn, triggered checking behaviour. This may initially be understood as goal-directed, safety-seeking behaviour in acutely psychotic paranoid patients. As one patient in this cohort reported, ‘I need to check everything is in place as my upstairs neighbour comes to steal my stuff’. Then, in patients achieving psychosis remission, we found checking severity to be significantly correlated with clozapine plasma levels, suggesting a role of clozapine in perpetuating checking as a non-goal-directed action or habit. Indeed, persistently impaired safety signalling has been described in OCD,^15^ limiting patients’ ability to assign safety valuations after verification.

Appreciating the distinct roles of psychosis and clozapine in OCS development is essential to understanding the apparent discrepancies in previous cross-sectional studies. The point prevalence of OCS (measured via questionnaire) may fluctuate according to the severity of the psychosis (Fig. 1, Table 2), clozapine load (Fig. 2), concomitant medications (e.g. antidepressants) or even the tools used to assess the symptoms. For instance, psychosis-driven, goal-directed checking might not be considered OCS but part of a delusion. Here we circumvented this risk by using a patient-reported OCS questionnaire.

Our hypothesised two-phase model of OCS development^9^ is based on cognitive neuroscience's framework of habit formation^11^ and shows the potential for embedding cognitive neuroscience into clinical practice. In our context, clozapine-treated patients in psychosis remission might experience checking compulsions as an antipsychotic-induced habit. A notable strength of our two-phase model is its integration of present results, previous studies and patients’ narratives, in which psychosis-induced goal-directed behaviour becomes habitual. This model is also flexible regarding the content of the repetitive behaviour, as we found checking whereas others reported washing as the most common compulsion.^16^ Therefore, it can accommodate other triggers, such as the influence of stressful events, cognitive dysfunctions or affective symptoms (not included in this study as they were not part of the *a priori* hypothesis).

A plausible mechanism for the persistence of repetitive behaviour following remission from psychosis may be clozapine's antagonism of 5-HT_2A_ and 5-HT_2C_ receptors. A decrease in serotonin neurotransmission causes perseveration in reversal learning tasks, the hallmark of cognitive inflexibility in compulsive behaviours such as OCD.^17^ In humans, dietary tryptophan depletion (which reduces serotonin in the brain acutely) promotes habitual rather than goal-directed control.^18^ Remarkably, a recent mouse-model study showed similar results. Mice receiving clozapine increased their grooming time significantly (as a proxy of compulsion). This behaviour then reverted on the administration of fluoxetine, a selective serotonin reuptake inhibitor. Interestingly, the same effects were seen in both wild-type and *Sapap3*-knockout mice (a well-known animal model of OCD), albeit with significantly greater intensity in the knockout mice, suggesting a genetic vulnerability to clozapine-induced habit formation.^19^

Nevertheless, it is also interesting to note that individual factors might increase vulnerability. For instance, 35 patients (17.9%) exhibited negligible OCS after 5 years or more on clozapine treatment. Variation in vulnerability was explored in this work, in which we suggest a distinct moderating effect of serotonergic and glutamatergic SNPs on the psychosis-to-obsession and obsession-to-compulsion pathways respectively (Supplementary material). We found evidence to support previous findings involving the glutamate pathway,^20^ such as SLC1A1. More importantly, we report the first indication of serotonin pathway involvement, as described in pure OCD. However, drawing firm conclusions about the role of specific variants will require work with larger samples. Nevertheless, our identification of several genetic variants in the serotonin pathway (SLC6A4, HTR2C) moderating the psychosis–obsession–compulsion pathway is notable, and may offer clues to future preventive or therapeutic approaches. The converging evidence from this and other studies indicates that interventions directed at enhancing serotonin function are crucial for the effective treatment of clozapine-induced OCS,^21^ as in pure OCD. Nevertheless, we did not specifically explore the effect of medication modifications on OCS severity, and further research is needed in this area.

A better understanding of the different phases of the phenomenon, in which compulsions arise as a by-product of florid paranoid psychosis and then perpetuated in predisposed subjects by clozapine’s anti-serotonergic action, may inform clinicians' therapeutic decisions.

## Supporting information

Fernandez-Egea et al. supplementary material 1 Fernandez-Egea et al. supplementary material

Fernandez-Egea et al. supplementary material 2 Fernandez-Egea et al. supplementary material

Fernandez-Egea et al. supplementary material 3 Fernandez-Egea et al. supplementary material

Fernandez-Egea et al. supplementary material 4 Fernandez-Egea et al. supplementary material

Fernandez-Egea et al. supplementary material 5 Fernandez-Egea et al. supplementary material

## Data Availability

The data that support the findings of this study, together with access to the code and CRD database, are available from the corresponding author on reasonable request.
