# Supplementary material for: The role of psychosis and clozapine load in excessive checking in treatment-resistant schizophrenia: longitudinal observational study
Source: Br J Psychiatry. 2024 May;224(5):164–9. doi: 10.1192/bjp.2024.30 (PMC11039551; doi:10.1192/bjp.2024.30)
Supplement: Fernandez-Egea et al. supplementary material 1 — Fernandez-Egea et al. supplementary material [file S0007125024000308sup001.docx]

**Supplementary table.** Multi-level moderation analysis of the genetic variants associated with the transitions from psychosis to obsession and obsession to compulsion.

| **gene** | **Subtype** | **N of subjects** | **gene moderates from psychosis to obsessions** | | **gene moderates from obsessions to compulsions** | |
| --- | --- | --- | --- | --- | --- | --- |
| *SCL6A4*  rs4795541 | SS | 10 | (reference) | (reference) | (reference) | (reference) |
|  | SL | 48 | 0.03 (-0.26, 0.32) | 0.835 | 0.13 (-0.33, 0.57) | 0.588 |
|  | LL | 36 | 0.28 (-0.02, 0.59) | 0.072 | -0.03 (-0.49, 0.44) | 0.91 |
| *SCL6A4*  rs25531 | AA | 82 | (reference) | (reference) | (reference) | (reference) |
|  | GA | 11 | 0.02 (-0.20, 0.24) | 0.865 | **0.41 (0.05, 0.77)** | **0.027** |
|  | GG | 1 | - | - | - | - |
| *SLC1A1*  Rs2228622 | GG | 31 | (reference) | (reference) | (reference) | (reference) |
|  | GA | 52 | 0.17 (-0.01, 0.34) | 0.072 | 0.09 (-0.21, 0.40) | 0.545 |
|  | AA | 11 | 0.09 (-0.16, 0.33) | 0.477 | **0.47 (0.06, 0.88)** | **0.027** |
| *GRIN2B*  rs890 | AA | 22 | (reference) | (reference) | (reference) | (reference) |
|  | AC | 45 | -0.04 (-0.24, 0.17) | 0.742 | -0.08 (-0.39, 0.24) | 0.632 |
|  | CC | 27 | 0.06 (-0.17, 0.28) | 0.616 | 0.14 (-0.19, 0.48) | 0.414 |
| *HTR2C*  rs3813928 | A or AA | 20 | (reference) | (reference) | (reference) | (reference) |
|  | GA | 4 | 0.50 (-0.08, 1.07) | 0.099 | -0.28 (-0.77, 0.22) | 0.28 |
|  | G or GG | 70 | 0.06 (-0.15, 0.27) | 0.586 | 0.05 (-0.30, 0.40) | 0.781 |
| *HTR2C*  rs1414334 | G or GG | 81 | (reference) | (reference) | (reference) | (reference) |
|  | GC | 2 | **2.91 (0.42, 5.39)** | **0.024** | 0.10 (-0.67, 0.88) | 0.802 |
|  | C or CC | 11 | 0.14 (-0.14, 0.41) | 0.343 | 0.18 (-0.17, 0.53) | 0.322 |
| *HTR2A*  rs6313 | T or TT | 17 | (reference) | (reference) | (reference) | (reference) |
|  | CT | 50 | 0.17 (-0.04, 0.37) | 0.111 | -0.09 (-0.45, 0.29) | 0.649 |
|  | C or CC | 27 | 0.20 (-0.05, 0.45) | 0.128 | -0.03 (-0.43, 0.39) | 0.901 |
| *HTR2A*  rs6314 | T or TT | 1 | - | - | - | - |
|  | CT | 14 | (reference) | (reference) | (reference) | (reference) |
|  | C or CC | 79 | -0.22 (-0.52, 0.06) | 0.133 | 0.21 (-0.18, 0.60) | 0.3 |
