# Supplementary figures and images for: The role of psychosis and clozapine load in excessive checking in treatment-resistant schizophrenia: longitudinal observational study
Source: Br J Psychiatry. 2024 May;224(5):164–9. doi: 10.1192/bjp.2024.30 (PMC11039551; doi:10.1192/bjp.2024.30)

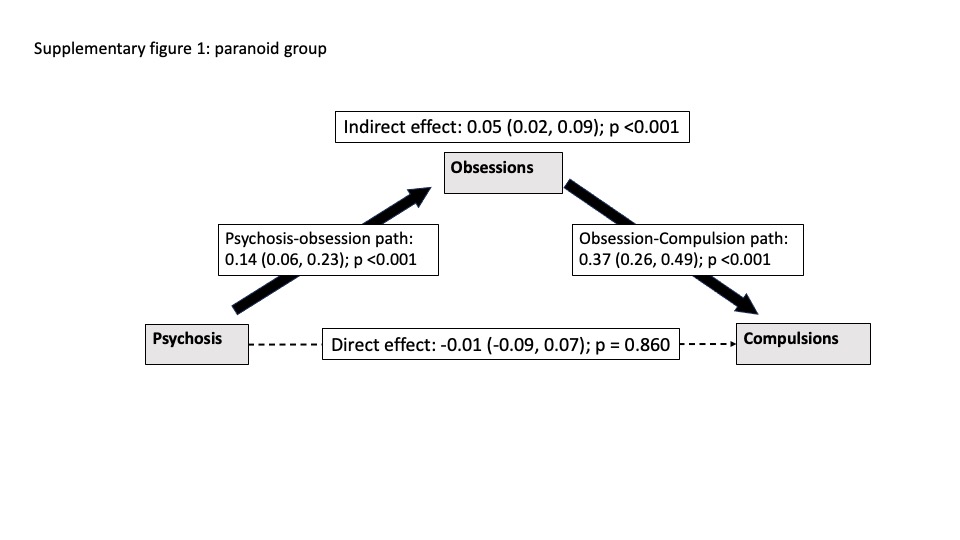

Supplement: Fernandez-Egea et al. supplementary material 2 — Fernandez-Egea et al. supplementary material [file S0007125024000308sup002.jpg]

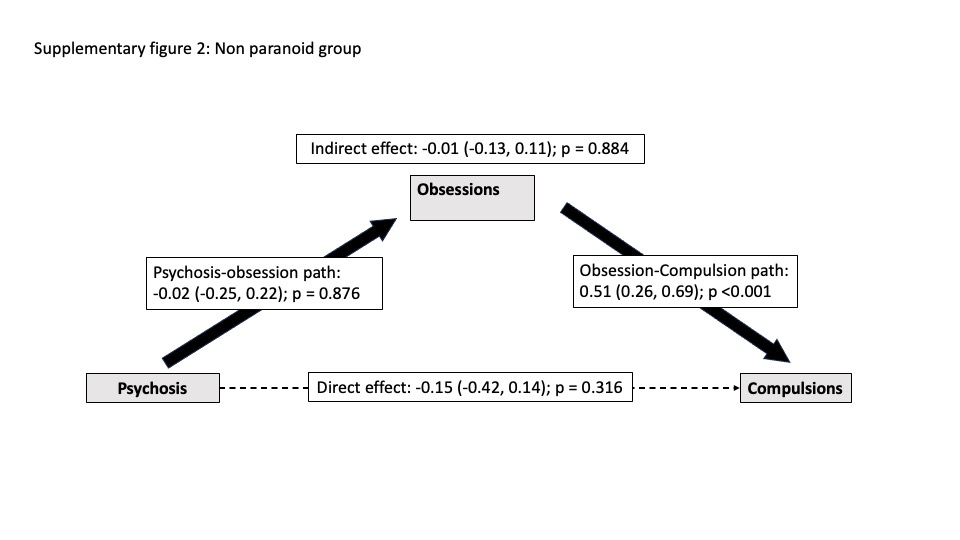

Supplement: Fernandez-Egea et al. supplementary material 3 — Fernandez-Egea et al. supplementary material [file S0007125024000308sup003.jpg]
