# Supplementary material for: The role of psychosis and clozapine load in excessive checking in treatment-resistant schizophrenia: longitudinal observational study
Source: Br J Psychiatry. 2024 May;224(5):164–9. doi: 10.1192/bjp.2024.30 (PMC11039551; doi:10.1192/bjp.2024.30)
Supplement: Fernandez-Egea et al. supplementary material 4 — Fernandez-Egea et al. supplementary material [file S0007125024000308sup004.docx]

**Table 1.**

**Sup Figure 1. OCS and psychosis among people with paranoid symptoms.** Mediation model for exploring causality. Psychosis was measured with the PANSS-positive subscale.

**Sup Figure 2. OCS and psychosis among people without paranoid symptoms.** Mediation model for exploring causality. Psychosis was measured with the PANSS-positive subscale.
