# Supplementary material for: The role of psychosis and clozapine load in excessive checking in treatment-resistant schizophrenia: longitudinal observational study
Source: Br J Psychiatry. 2024 May;224(5):164–9. doi: 10.1192/bjp.2024.30 (PMC11039551; doi:10.1192/bjp.2024.30)
Supplement: Fernandez-Egea et al. supplementary material 5 — Fernandez-Egea et al. supplementary material [file S0007125024000308sup005.docx]

**Supplementary material**

Supplementary Table. Rates of obsessive–compulsive disorder (OCD) by psychosis severity at baseline.

|  |  | Psychosis Remission | | total |
| --- | --- | --- | --- | --- |
|  |  | **No** | **Yes** |  |
| OCD Overall severity* | *Negligible (0–4)* | 18 (62.1%) | 11 (37.9%) | 29 |
|  | *Moderate (5–21)* | 66 (64.1%) | 37 (35.9%) | 103 |
|  | *Severe (>21)* | 51 (81.0%) | 12 (19.0%) | 63 |
|  | *Total* | 135 | 60 | 195 |
|  |  |  |  |  |
| Excessive Checking | *No (<5)* | 75 (68.2%) | 35 (31.8%) | 110 |
|  | *Yes (>5)* | 60 (70.6%) | 25 (29.4%) | 85 |
|  | *Total* | 135 | 60 | 195 |

*OCD overall severity was calculated using the total OCI-R score. Excessive checking refers to the OCI-R checking subscale (3 items). Psychosis remission refers to all PANSS positive subscale items being scored <3.
